# Effect of bacteria type and sucrose concentration on levan yield and its molecular weight

**DOI:** 10.1186/s12934-017-0703-z

**Published:** 2017-05-23

**Authors:** Álvaro González-Garcinuño, Antonio Tabernero, José Mª Sánchez-Álvarez, Miguel A. Galán, Eva M. Martin del Valle

**Affiliations:** Department of Chemical Engineering, University of Salamanca, Plza. Los Caidos s/n, 37007 Salamanca, Spain

**Keywords:** Levan, Molecular weight, Kinetics, Byproducts

## Abstract

**Background:**

Levan has been traditionally produced from microorganism. However, there is a continuous effort in looking for new strains that improve levan production yield and uses alternative sugar sources for growth. Despite having a wide range of data about levan yield, there are not papers which allow controlling molecular weight, and that plays an essential role for further applications.

**Results:**

The effect of the sucrose concentration on levan yield (and its molecular weight) from *Bacillus atrophaeus* and *Acinetobacter nectaris* (Gram positive and Gram negative respectively) was studied in this work. It was found that *A. nectaris* growth (from 3 to 1.5 g L^−1^ in 40 h) and its levan production (from 3 to 1.5 g L^−1^) decreases by increasing sucrose concentration (best results at a concentration of 120 g L^−1^) whereas *B. atrophaeus* growth (3.5 g L^−1^ in 30 h) and its levan production (also 3.5 g L^−1^) were not affected by modifying that parameter. Levan molecular weight from *A. nectaris* decreases by increasing sucrose concentration (from 8000 to 2000 kDa) whereas levan molecular weight from *B. Atrophaeus* remains always around 50 kDa. By performing a kinetic study, it was shown that *A. nectaris* growth follows a substrate-inhibition model, whereas Monod equation provided a good fit for *B. atrophaeus* growth. Finally, wastes from orange juice industry were used as a medium culture to cultivate those microorganism, obtaining good results with *B. atrophaeus* (growth 3 g L^−1^ in 30 h).

**Conclusions:**

Levan production kinetics was determined and compared between different bacteria types.

**Electronic supplementary material:**

The online version of this article (doi:10.1186/s12934-017-0703-z) contains supplementary material, which is available to authorized users.

## Background

Levan is a homopolysaccharide of fructose units which is produced by some plants and microorganisms. These organisms have an enzyme called levansucrase (EC.2.4.1.10), which breaks the sucrose bond between glucose and fructose, and after that, polymerizes these fructoses linked β (2 → 6). Levan is considered as an exopolysaccharide (EPS), because it is synthesized outwardly the cell. Levansucrase is translated from its respective mRNA in cell cytosol and is secreted to bacterial peptidoglycan wall at acid pH (5–6) where it catalyzes the reaction described above [1].

Levan is a non-typical biopolymer because it is formed by furanoses units (fructose), whereas other biopolymers are formed by pyranoses residues. This fact could have an incredibly interest for different applications such as industry, cosmetics, medicine or nanotechnology [2].

Levan has been traditionally obtained from microorganisms. *Zymomonas mobilis,* a Gram negative bacterium, is considered the most common microorganism for that matter. However, *Z. mobilis* produces ethanol at the same time, so a subsequent step is needed for levan purification. The other well-known bacterium for levan production is *Bacillus subtilis*, which has been extensively studied. Recently, new genera like *Hallomonas* or *Pseudomonas* have been used for levan production [3, 4]. In total, more than 50 studies have been performed with the aim of analyzing bacteria for levan production [1].

Those studies were focused on optimizing the medium culture in terms of pH, temperature, use of substrates, etc. As an example, Silbir et al. [5] used *Z. mobilis* strain B-14023 for levan production. These authors presented yeast extract as the most promising nitrogen source for levan production. Another example is the study published by Ing-Lung et al. [6], where temperature, culture time and pH are considered for levan production with *B. subtilis* (natto). Ing-Lun et al. [7] also studied the effect of sucrose concentration on levan production, obtaining the best results between 200 and 300 g L^−1^. However, none of these articles took into account the effect of the different medium culture in the polymer properties, such as molecular weight.

Nonetheless, there is not a study that explains the role of the bacteria type in levan production (e.g. differences between Gram negative and Gram positive bacteria) and although there are some articles reporting the kinetics of the levan production [8], to the best of our knowledge those kinetics have not been mathematically modelled and as a consequence they have not been fully investigated.

In addition, some studies such as Nicholson et al. [9] reported an important link-up between molecular weights and some polymer properties like melt viscosity, tensile strength, resistance to heat and corrosion properties. These properties could be really interesting for the applications described above such as biomedicine or food. As an example, levan antitumour activity depends on the molecular weight, obtaining a maximum antitumour inhibition for a viscosity molecular weight around 210,000 kDa [10]. Moreover, Elvassore et al. [11] suggested that higher PEG–PLA molecular weight of polymer PEG–PLA, produces a worse drug encapsulation efficiency as well as affecting the drug release. Specifically, a low polymer molecular weight involves a slow and constant drug release.

Furthermore, it is essential to investigate new trends for levan production in order to reduce costs. One of the possible options is the use of cheaper sources as alternative substrates. As an example, Roberto de Oliveira et al. [12] used some regional residuals (sugar cane syrups and sugar cane molasses) as a carbohydrate sources up to a sucrose concentration of 250 g L^−1^ to produce levan. However, wastes from fruit industry (that can be a proper alternative) have not been used yet as an alternative to protein sources (such as yeast extract).

Based on the above-mentioned facts, the main aims of this article are to study the effect of the different type of bacteria and the sucrose concentration on levan production and its molecular weight as well as modelling the kinetics involved in this biopolymer production process. Besides, a new substrate source (a food industry waste) is tested as an alternative culture medium.

In order to do that, two new strains (*Bacillus atrophaeus* and *Acinetobacter nectaris*), which have not been used before for levan production, were selected for this study. One of them is a Gram positive bacteria and the other is a Gram negative. Therefore, by using new experimental data and by performing a comparison of our results with some results from literature, the effect of the bacteria type on the polymer molecular weight may be explained. These strains were, in addition, selected in order to find new strains for producing levan as well as for identifying if an original Spanish isolated bacterium (*A. nectaris*) can be used for that purpose.

Moreover, the effect of the sucrose concentration in levan yield and its molecular weight was studied by modifying the amount of sucrose in the culture medium. Growth and levan yield results were in addition mathematically modelled in order to define the kinetics type followed by the studied bacteria. Finally, wastes from the orange juice industry were tested as an alternative source of proteins for producing levan, indicating the possibility of using food industry residuals to grow microorganisms for biopolymer production.

## Methods

### Microorganisms and culture medium

Two bacteria strains were selected from Spanish microorganism collection culture (CECT). Specifically, *B. atrophaeus* (CECT 0038) as a Gram negative bacteria and *A. nectaris* (CECT 8127) as a Gram positive bacterium. The last one was isolated from nectar in wild Mediterranean insect-pollinated plants at Doñana Park in Huelva (Spain).

Both strains were cultured in flask recipients (volume 250 mL), using the following culture media: 7 g L^−1^ yeast extract, 2.5 g L^−1^ K_2_HPO_4_, 1.6 g L^−1^ NH_4_SO_4_, 0.4 g L^−1^ MgCl_2_. After that, the pH was adjusted between 5.5 and 6 (with HCl 1 M). Experiments were carried out at 30 °C and a stirring speed 150 rpm [6].

Furthermore, in order to reduce the medium cost, a byproduct from the juice industry (juice pulp after oranges squeezing process) was used to cultivate the previous microorganisms. That pulp was heated at 90 °C in water in order to extract nutrients from it, and after that, that liquid was filtered to remove the remaining solids. A source of inorganic nitrogen (ammonium nitrate at 1 g L^−1^) and sucrose (120 g L^−1^) were added to that liquid in order to achieve all the required nutrients.

Since the composition of the juice pulp is unknown, that waste was previously analyzed by elemental microanalysis in order to know its chemical composition in carbon, hydrogen, nitrogen, oxygen and sulfur. This analysis procedure is given in “Levan molecular weight determination”.

### Biomass determination and levan production

Biomass could be determined by following the solids in suspension method proposed by Xin et al. [13]. This spectrophotometric technique consists of measuring the absorbance of the biomass at 650 nm. In this context, the following equations were obtained to calculate the biomass for both strains (Eqs. 1, 2):

1 $${\text{For }}A. \, nectaris{:\text{ y }} = \, 0.1091{\text{x }}{-} \, 0.0216$$

2 $${\text{For }}B. \, atrophaeus{:\text{ y }} = \, 0.1049{\text{x }}{-} \, 0.0089$$

where y is the absorbance at 650 nm, and x is biomass concentration expressed in mg/mL.

Using these equations, biomass was measured each 8 h in order to control cell density and microorganism growth.

At the same time, levan could be estimated by the spectrophotometric method proposed by Vigants et al. [14] at 400 nm. This technique takes advantage of the change of turbidity (at 25 °C) in the culture medium due to the levan formation. The procedure is based on the extraction of an aliquot from the flask (each 8 h) that must be centrifuged at 10,000 rpm during 10 min to eliminate biomass. After that, the absorbance was measured at 400 nm. As a result, an equation (Eq. 3) was obtained to determine the levan concentration in our culture medium:

3 $${\text{y }} = \, 0. 1 6 4 5 {\text{x }}{-} \, 0.0 3 5$$

where y is the absorbance at 400 nm, and x is levan concentration expressed in mg/mL.

Furthermore, the amount of glucose consumed for levan synthesis could be estimated using the Eq. 4 [15].

4 $${\text{M}}_{\text{glucose}} = \,\left( {180/162} \right)\cdot{\text{ M}}_{\text{levan}}$$

### Biomass recovery and levan isolation

When the culture reaches its stationary phase, biomass is collected and extracted from the medium culture by centrifugation at 10,000 rpm during 10 min. This biomass is discarded, since the levan will be secreted in the supernatant phase. With the aim of extracting and isolating the biopolymer, the following process will be used:

Firstly, pH was adjusted between 9.5 and 10.5 with KOH 0.1 M in order to stop the enzymatic reaction for levan production. After that, 1 mL CaCl_2_ (1% w/w) was added each 20 mL of supernatant [4], following by the addition of ethanol 96% (v/v) in proportion ethanol: supernatant 3:1. Finally, the resulting mixture is frozen at −20 °C. After 25 h, the levan precipitates and by a centrifugation process (6000 rpm during 10 min) the polymer is isolated.

After that, a dialysis process was performed to purify the polymer. Basically, levan was dissolved in deionized water and the dissolution was dialyzed against ultrapure water (ultrapure water was changed two times after 24 h) by using cellulose membranes (Orange Scientific, Belgium) with porous size (12–14 kDa). This porous size was chosen with the aim of removing low molecular weight substances such as proteins, nucleic acids or other organic compounds that could be presented in culture media.

The solution was collected after dialysis, and was lyophilized by Telstar Lyophilizaer at −55 °C and 0.020 bar in order to remove water and obtain the polymer as a solid product.

### Polymer structure determination

Infrared spectrum (IR) and nuclear magnetic resonance (NMR) were carried out in order to determine the nature of the polymer extracted from culture broth.

FT-IR spectra were recorded in a Perkin-Elmer Spectra ONE instrument, using KBr pellets; 32 spectra (recorded with a nominal resolution of 4 cm^−1^) were averaged to improve the signal-to-noise ratio.

Nuclear magnetic resonance spectra were recorded on a 200 and 400 MHz (1H) and 50 and 100 MHz (13C) spectrometers by using a Varian Mercury 200 MHz. FTIR spectra were recorded as films. HRMS spectra were recorded by using Q-TOF using electrospray ionization.

### Elemental microanalysis of biomass and orange wastes

In order to define the elemental composition of some substances (biomass or juice byproducts), the analysis of those products was performed by using a modified method from Pregl and Dumas (dynamic flash combustion). This method establishes a relative proportion for Carbon, Hydrogen, Nitrogen and Sulfur; determining Oxygen by difference.

Samples are encapsulated into a tin or silver vial and oxygen is injected into the vial. After that, the vial is placed in an oven at high temperature to produce combustion. Products from this combustion are: CO_2_, H_2_O, NO_x_ and SO_x_. These gases are transported along a pipe using Helium as carrier gas, finishing into an oxidation–reduction pipe. Finally, gases flow through a non-dispersive infrared detector with the aim of determining H_2_O, CO_2_ and SO_2_ concentration. N_2_ is measured by thermal conductivity.

### Levan molecular weight determination

Molecular weight was determined by static light scattering (SLS) with Zsizer Nano (Malvern instruments). This method is a non-invasive technique that can be used to characterize molecules in a solution. Particles in the sample are illuminated by a light source such as laser, with the particles scattering the light in all directions. MW is determined by measuring the sample at different concentrations and applying the Raleigh equation (Eq. 5), which describes the intensity of light scattered from a particle in solution.

5 $$\frac{K \cdot C}{{R_{\theta } }} = \left( {\frac{1}{{M_{w} }} + 2A_{2} C} \right)P_{\theta }$$

where K is an optical constant which depends on laser wavelength, solvent refractive index and the differential refractive index increment. R_θ_ is the Raleigh ratio and P_θ_ is the angular dependence of the sample scattering intensity. Mw is molecular weight for the sample. A_2_ is the second virial coefficient; and C is the concentration. To determine the second virial coefficient and the molecular weight, samples with different polymer concentrations were analyzed by SLS. MW was determined as the interception of Y-axis whereas the slope is related with 2nd virial coefficient.

It is important to specify that this technique needs a standard compound with a well-known behavior in terms of SLS molecular weight determination. In this context, toluene was chosen because it is the most used compound for this purpose.

### Growth kinetics equation determination

Kinetics for microbial growth could be estimated using an unstructured kinetic model, where the specific growth rate (μ) is determined by using the differential Eq. 6.

6 $$\frac{dx}{dt} = (\mu - D)x$$

where dx/dt is the biomass increment with time, x is biomass concentration at each time, and μ is the specific growth rate.

In this case, D (dilution rate) is considered null because the experiments were performed at batch scale.

Furthermore, biomass results were adjusted to models based on Monod Model [16]. Product rate is calculated following the Eq. 7.

7 $$R_{p} = \frac{1}{x}\frac{dp}{dt}$$

where Rp is product rate, x biomass density, and dp/dt, the variation in product concentration with the time.

Moreover, it was studied if levan production could be adjusted to Luedeking–Piret model [17] (product formation is associated to microbial growth). Luedeking–Piret model is described in Eq. 8.

8 $$Rp = \upbeta_{xp} \cdot \mu + m_{p}$$

where $\beta_{xp}$ relates product with biomass, m_p_ is the maintenance coefficient (or ATP coefficient), Rp is product rate and μ is the specific growth rate

On the other hand, stoichiometric coefficients were determined in order to complete the reaction general equation (Eq. 9):

9 $$CH_{1.83} O_{0.91} + \alpha O_{2} + \beta (NH_{4} )_{2} SO_{3} \to \gamma CH_{x} O_{y} N_{z} + \delta CH_{1.77} O_{0.88} + \varepsilon CH_{2} O + \omega CO_{2}$$

this reaction corresponds to:

Sucrose + oxygen + ammonium sulfate → biomass + levan + (glucose + fructose) + carbon dioxide

It is assumed that yeast extract is only used for essential amino acids uptake, and it has no contribution in mass balance.

## Results

Results are organized in the following way. Firstly, “Influence of substrate concentration in biomass growth and its kinetic study” and “Influence of substrate concentration in levan production and its kinetic study” describe the effect of the substrate concentration in biomass growth and levan production. The kinetic study is also included in each respective section.

Levan characterization is explained in “Levan characterization”, whereas the effect of the sucrose concentration on the molecular weight is studied in “Influence of substrate concentration in levan molecular weight for both strains”. On the other hand, experimental and kinetics studies of how glucose inhibits the levan yield are performed in “Levan inhibition with glucose”. Finally, the use of a waste product as a medium culture for producing levan is proposed in “Wastes from juice factories as a substrate for cultivating both strains”.

### Influence of substrate concentration in biomass growth and its kinetic study

Different concentrations of sucrose were added to the microorganism culture medium. Specifically, experiments were carried out with 120, 150, 180, 210, 240 and 270 g L^−1^ sucrose for both strains. Those concentrations have been used previously in different articles [7, 12]. Biomass and levan were determined each 8 h following the methods already described in the experimental section. The pH remained between 5.5 and 6 because an acid medium is needed to secrete the levansucrase to the bacterial cell wall.

**Fig. 1:**
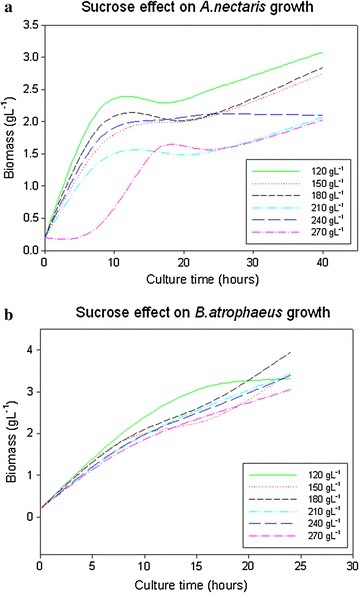
Sucrose concentration effect on microbial growth. **a** Effect on *A. nectaris*. **b** Effect on *B. atrophaeus*

Growth results are shown at Fig. 1. Graph A belongs to biomass growth for *A. nectaris* and Graph B belongs to *B. atrophaeus* results.

As can be observed in Fig. 1a (for *A. nectaris)*, biomass proliferation decreased with the sucrose (from 3.0 with 120 g L^−1^ of sucrose to 2.0 g L^−1^ with more than 180 g L^−1^ of sucrose). However, for *B. atrophaeus*, the sucrose did not produce a significant effect on the microorganism growth (Fig. 1b) and the biomass growth ranged from 3.0 to 4.0 g L^−1^. It can be concluded that the best sucrose concentration was around 120 g L^−1^ for *A. nectaris* (biomass growth around 3 g L^−1^ in 30 h) whereas the optimum value was around 180 g L^−1^ for *B. atrophaeus* (biomass growth around 4 g L^−1^ in 30 h).

After determining biomass proliferation, the microorganism growth kinetics was studied. As was described before, *A. nectaris* may follow a typical substrate-inhibition kinetic (Eq. 10 [18]).

10 $$\mu = \upmu_{\hbox{max} } \frac{S}{{\frac{{S^{2} }}{Ki} + S + K_{s} }}$$

The following kinetic coefficients were obtained by fitting experimental data-theoretical data: $\mu_{\hbox{max} }$: 3.207 h^−1^, Ks: 2068 g L^−1^, Ki: 9.73 g L^−1^.

The classical Monod model was used for fitting the experimental data with *B. atrophaeus* (without sucrose inhibition). A good correlation coefficient (R^2^ > 0.90) was obtained, whereas the kinetic parameters are: $\mu_{\hbox{max} }$: 0.107 h^−1^, Ks: 5.48 g L^−1^.

### Influence of substrate concentration in levan production and its kinetic study

**Fig. 2:**
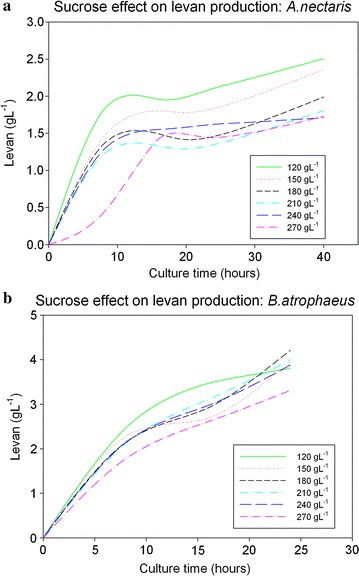
Sucrose concentration effect on levan synthesis. **a** Effect on *A. nectaris*; **b** effect on *B. atrophaeus*

While cultivating both bacteria, the levan produced was determined each 8 h, using the spectrophotometric method described above. Results for both strains are illustrated in Fig. 2.

Levan production and biomass proliferation follow the same tendency. Levan production from *A. nectaris* decreases by increasing sucrose concentration (from 2.5 at 120 g L^−1^ of sucrose to 1.5 with 270 g L^−1^ of sucrose). On the other hand, there was not a clear effect of sucrose concentration on *B. atrophaeus* levan yield (between 3.0 and 4.0 g L^−1^ for all sucrose concentrations in the medium). That is, sucrose concentration inhibited levan production in Gram negative bacteria, but it had no effect on Gram positive bacteria.

Assuming that levansucrase follows the well-known Michaelis–Menten kinetic equation [19]; parameters *Vmax* and *Km* were determined for both strains by fitting experimental to theoretical data. For *A. nectaris*, *Vmax* and *Km* vales are: 0.061 g L^−1^ h^−1^, and 66 g L^−1^, respectively. For *B. atrophaeus*, *Vmax* and *Km* values are: 0.109 g L^−1^ h^−1^ and 54 g L^−1^. These results agreed with the inhibition hypothesis for Gram negative strains. *B. atrophaeu*s can produce levan two times faster than *A. nectaris*, and substrate specificity was higher for *B. atrophaeus* than for *A. nectaris* (the lowest *Km* was obtained for *B. atrophaeus*).

With the previous results, it is possible to analyze if levan production is associated with biomass growth. This fact, which has not been studied previously in the literature, must be taken into account for a scale-up. Firstly, it was assumed that levan is associated with biomass proliferation, following Luedeking–Piret equation (Eq. 8).

Luedeking–Piret equation was used for fitting the experimental data for each sucrose concentration for both strains, obtaining the following parameters (Table 1)

**Table 1:** Coefficient determination for βxp

*A. nectaris*			*B. atrophaeus*		
[Sucrose] (g/L)	βxp	M_p_	[Sucrose] (g/L)	βxp	M_p_
120	6.079	−5E−17	120	1.3427	−0.0004
150	6.079	+2E−16	150	1.3915	−0.0071
180	6.079	+1E−16	180	1.3032	−0.0069
210	6.079	−6E−17	210	1.4255	−0.0052
240	6.079	+2E−16	240	1.4133	−0.0059
270	6.079	−8E−17	270	1.2588	−0.0007
Medium value	6.079	+5.2E−17	Medium value	1.3558	−0.0044

Finally, in order to complete the reaction general equations, stoichiometric coefficients were determined as it was explained in kinetics section. For performing mass balances calculation, two initial conditions were determined experimentally: βxp that can be calculated as *δ*/*γ*, that can be calculated from Vigants experiments—Eq. (4). In our case, *δ* = 0.562 Cmol levan/Cmol sucrose, whereas βxp is taken for each strain after modelling experimental data with Luedeking–Piret equation. Stoichiometric coefficients for both bacteria are indicated in Table 2.

**Table 2:** Stoichiometric coefficients determined from experimental data (expressed in Cmol substance/Cmol sucrose)

	*A. nectaris*	*B. atrophaeus*
*α*	0.02	≈0
β	0.0105	0.047
γ	0.105	0.470
δ	0.562	0.562
ε	0.301	0.180
ω	0.033	≈0

Respiration quotient (RQ), which is defined as quotient between $\alpha$ and $\omega$, could be determined, with the following results: for *A. nectaris* RQ = 1.69 and for *B. atrophaeus* it was not possible to calculate because both values are aprox. 0.

### Levan characterization

**Fig. 3:**
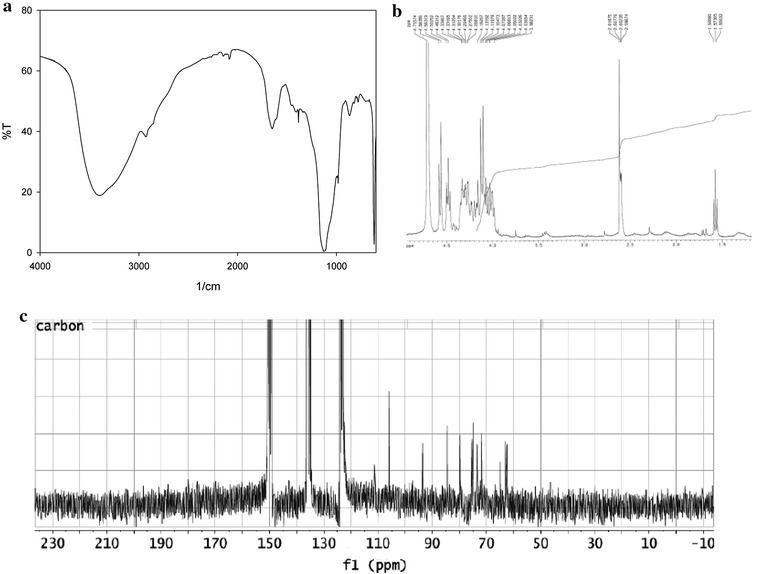
Characterization of levan obtained. **a** Infrared spectrum, **b** H-MRN, **c** C-MRN

After levan isolation and purification, several analytical techniques were used to check polymer structure. Figure 3 shows spectrums for levan extracted (A: infrared spectrum, B: H-NMR, C: C-NMR).

These figures confirm the production of levan from the selected strains due to the similarities between the obtained IR spectrum and the different spectra from literature [4, 21]. Same similarities were found for the nuclear magnetic resonance (NMR) spectra. The six ^13^C NMR characteristic levan broad signals were at the different ppm (62.3, 64.7, 75.1, 75.5, 79.8, 105.9). Those signals were described in literature [20].

### Influence of substrate concentration in levan molecular weight for both strains

**Fig. 4:**
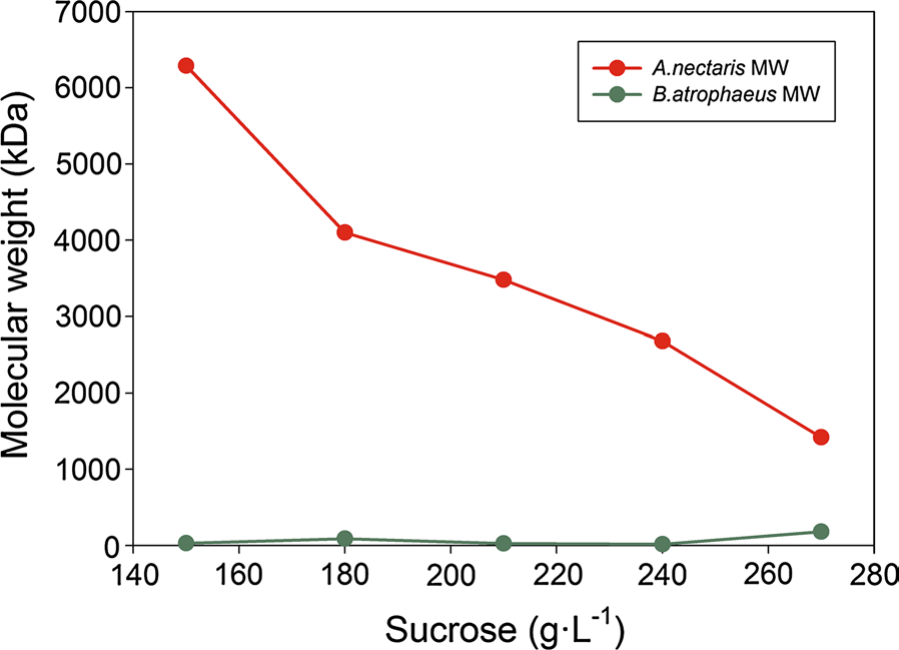
Levan molecular weight depending on sucrose initial concentration

Levan molecular weight for each strain and at the different sucrose concentrations was determined by static light scattering (SLS). Figure 4 shows the effect of the sucrose concentration on levan molecular weight.

As can be observed, several differences can be found for both strains. *A. nectaris* molecular weight was higher than the *B. atrophaeus* molecular weight. *A. nectaris* levan molecular weight ranged 1000–6000 kDa, whereas *B. atrophaeus* produced a levan with a molecular weight ranging from 15 to 90 kDa. Furthermore, an equation relating *A. nectaris* molecular weight with sucrose concentration (g L^−1^) was determined (Eq. 11).

11 $$y = - 37.2x + 11406$$

where *y* is the molecular weight in kDa, and *x* is the sucrose concentration in g L^−1^.

### Levan inhibition with glucose

Based on the previous results, sucrose can inhibit biomass and levan production in Gram negative bacteria (*A. nectaris*). However, it is unclear the mechanism of that inhibition. It is well-known [21] that glucose inhibits invertase in *Saccharomyces cerevisae*. Invertase (EC.3.2.1.26) is an enzyme which catalyzes the sucrose breaking into glucose and fructose, but it does not polymerize that fructose.

**Fig. 5:**
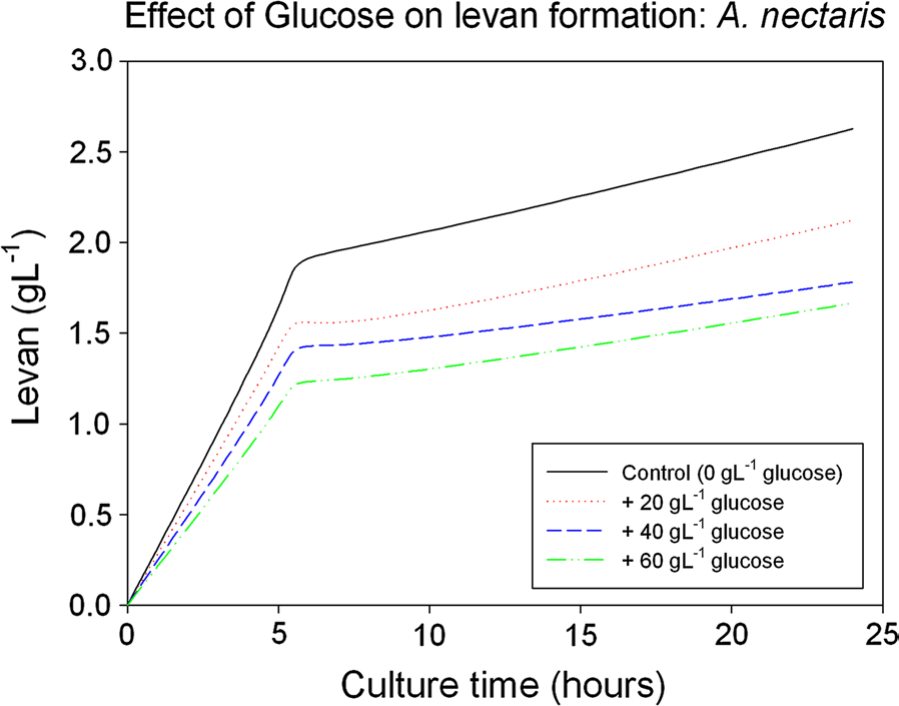
Effect of glucose on levan formation from *A. nectaris*

Some experiments were performed in order to prove if levansucrase follows a similar mechanism for inhibition. Different concentrations of glucose were added to *A. nectaris* culture medium and levan production was determined. Results are sown in Fig. 5.

As can be seen in Fig. 5, levan production decreased by increasing glucose concentration (ranging from 2.0 g L^−1^ without glucose in the medium to around 1.2 with 60 g L^−1^ of glucose in the medium).

Based on the previous experiments, an equation was obtained to predict the polymer formation depending on the glucose concentration (Eq. 12).

12 $$\frac{dp}{dt} = - 0.0018 [I] + 0.3255$$

where [I] is glucose concentration in g glucose per liter. More data concerning these calculations are included in the Additional files 1, 2.

### Wastes from juice factories as a substrate for cultivating both strains

**Fig. 6:**
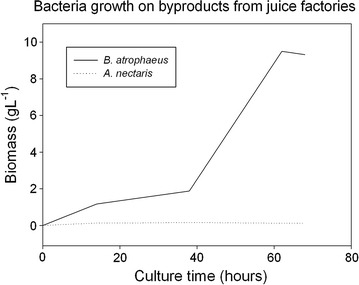
Bacteria growth on byproducts from juice factories

*Acinetobacter nectaris* and *B. atrophaeus* were cultivated with wastes from an orange juice factory. The composition of this residual was analyzed with the aim of determining its molecular formula. After performing the analysis, the molecular formula was CH_1.76_O_0.90_N_0.04_. Due to the low nitrogen value, ammonium nitrate was used as a nitrogen source. That source has been described previously as good nitrogen source for bacteria growth [22]. Also, sucrose was added at 120 g L^−1^ because that concentration provided the highest yield without inhibiting microorganism growth and polymer production. Figure 6 illustrates the results of cultivating both bacteria with this culture medium.

As can be observed in Fig. 6, *B. atrophaeus* grew in the new culture medium by using wastes from orange juice factories, with a similar growth rate in comparison with the results obtained for the same strain with the common medium (Fig. 1). However, *A. nectaris* did not grow when wastes are used because these residuals did not provide enough nutrients for that strain. Further studies must be done in order to determine which nutrients have to be added to grow *A. nectaris*.

After that, *B. atrophaeus* biomass was recovered and the levan was isolated and purified, producing 24.2 g levan L^−1^. This value is similar to the result obtained with the common culture medium.

Levan molecular weight obtained from *B. atrophaeus* was 200 kDa. This value is approximately two times the levan molecular weight that was synthesized with the common medium. Nevertheless, this molecular weight is still lower than levan molecular weight from *A. nectaris*.

## Discussion

### Substrate effect on biomass yield

**Fig. 7:**
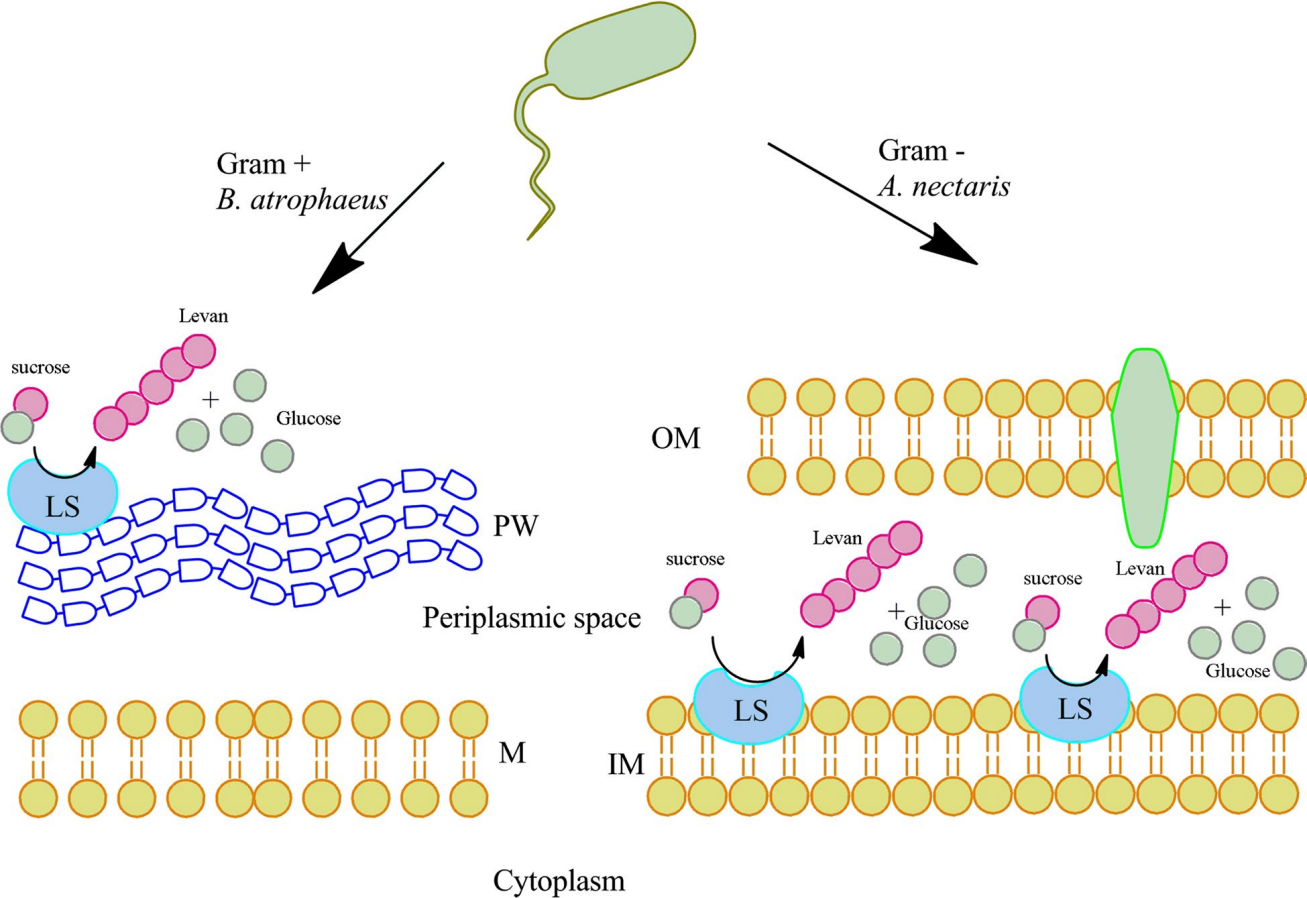
Schematic vision of method proposed. In Gram negative bacteria, sucrose and products are imbibed in periplasmic space, and there is mass transfer opposition for go away to culture broth. This fact does not occur with Gram positive bacteria, where enzyme is directly in contact with culture broth. *M* membrane, *IM* inner membrane, *OM* outer membrane, *PW* peptidoglycan wall, *LS* levansucrase

The results concerning the effect of substrate (Fig. 1) can be explained depending on the different bacteria type. *A. nectaris* is a Gram negative bacterium and as consequence the levansucrase can be located at their periplasmic space. On the other hand, *B. atrophaeus* is a Gram positive strain, so sucrose and enzyme cannot be in contact for a long time, due to the existence of a peptidoglycan wall. Therefore, the inhibition in *A. nectaris* was produced due to the accumulation in its periplasmic space. This contact can produce enzyme inhibition by substrate. This possible mechanism is illustrated in Fig. 7.

These results agree with the ones that have been proposed previously by Senthilkimar and Gunasekaran [23] with a classical Gram negative bacterium (*Zymomonas mobilis*). Furthermore, previous results with other Gram positive bacterium (*Bacillus subtilis*) show that biomass proliferation is independent from sucrose concentration [6].

### Substrate effect on levan yield

The results concerning levan production also confirm the hypothesis described in Fig. 7, where sucrose in periplasmic space does not only inhibit biomass growth but also levan production.

In comparison with literature, it is possible to find strains that produce a similar amount of levan. As an example, *B. subtilis (natto*) ATTCC 7058 produced 3.6 g L^−1^ in 21 h [7], 1–2 g L^−1^ of levan was produced after cultivating *Pseudomonas fluorescens* in 30 h [4] whereas only around 1 g L^−1^ was obtained after extracting levan from *Halomonas* sp. *AAD6* [3].

From kinetics results (observed in Table 1), it can be concluded that levan production is associated to the biomass growth. *A. nectaris* showed a better ratio product-biomass than *B. atrophaeus*, meaning that *A. nectaris* produced six times more levan for each biomass gram than *B. atrophaeus*. This fact suggested *A. nectaris* as an effective bacterium for levan production due to its high levan production for a lower biomass proliferation. Obtaining high amounts of levan without requiring a great biomass growth is a good strain quality for a future scale-up process. Moreover, results from Table 2 (RQ) confirm that product formation in *A. nectaris* was more efficient than in *B. atrophaeus*.

### Molecular weight

As it has been mentioned in results chapter, levan molecular weight from *A. nectaris* can be controlled by sucrose concentration. However, results showed that initial sucrose concentration did not modify levan molecular weight obtained from *B. atrophaeus*. This fact can be explained due to the cell wall and membrane structure. Levan polymerization in *A. nectaris* is located the periplasmic space, and as a consequence the polymer experience some difficulties to cross this space towards the culture broth. Therefore, the polymer could remain in the plasmatic membrane and can incorporate new fructose unit, increasing its molecular weight. The previous results highlighted a new possibility for controlling polymer molecular weight by modifying sucrose concentration in the medium culture.

### Glucose inhibition

Results from glucose experiments suggest that glucose may be one of the factors that can be involved in levan inhibition for Gram negative bacteria, and explain why an increase of sucrose in the medium (and as a consequence more glucose) inhibits levan production for this type of bacteria. In this context, Eq. [12] can be used as a tool to determine levan and biomass yield depending on the glucose concentration.

### Waste from juice factories

Results from experiments that considered wastes from juice factories, highlight that there is a possibility of reducing the culture medium cost by using an alternative and inexpensive medium, such as residuals from the juice factories.

As a matter of fact, after performing some economic calculations, it is possible to reduce the cost of the medium culture (without considering sucrose) up to an 86%. This reduction ranges from 604 € m^−3^ (rich medium) to 87 € m^−3^ (new substrate source). If sucrose price is included, reduction reaches 10% (from 5000 to 4482 € m^−3^). Considering a four days process, the cost reduction is 129 € m^−3^ day^−1^. More details regarding these calculations are included in Additional file 2.

Although several works have studied the influence of different parameters (pH, temperature, substrate types and concentration) in levan production, the use of wastes from fruit factories has not been described yet.

## Conclusions

The effect of sucrose concentration and the bacteria type on levan yield and its molecular weight has been studied in this work. Two new strains (*A. nectaris* and *B. atrophaeus*) were selected and cultivated to synthesize levan. Specifically, they respectively produce around 3 and 3.5 g L^−1^ of polymer. Results indicate that an increase of sucrose concentration decreases levan yield and molecular weight (from 8000 to 2000 kDa) for *A. nectaris* strain, whereas it does not produce a significant effect on *B. atrophaeus* growth and levan molecular weight (more or less 50 kDa at all the investigated conditions). The best sucrose concentrations were 120 g L^−1^ for *A. nectaris* and around 180 g L^−1^ for *B. atrophaeus*. The difference in those results is explained by taking into account the existence of peptidoglycan wall in Gram positive bacteria. Besides, by modelling growth kinetics, it can be concluded that *A. nectaris* follows a substrate-inhibition kinetics controlled by glucose whereas *B. atrophaeus* follows a typical Monod model. Finally, *B. atrophaeus* is able to grow (3 g L^−1^ in 30 h) by using a medium culture with wastes from orange juice industry as an alternative culture medium, indicating the possibility of using wastes for growing bacteria with the aim of producing levan.

## Additional files

**Additional file 1.** Additional material A.

**Additional file 2.** Additional material B.

## Abbreviations

EPS: exopolysaccharide
PEG–PLA: polyethylene glycol–polylactic acid
CECT: Spanish microorganism collection culture
IR: infrared spectrum
NMR: nuclear magnetic resonance
FTIR: fourier transform infrared spectrum
SLS: static light scattering
MW: molecular weight
µ: specific growth rate
D: dilution rate
x: biomass concentration
t: time
R_p_: product rate
p: product concentration
β_xp_: Luedeking–Piret coefficient
m_p_: maintenance coefficient
Ks: monod constant
Ki: inhibition constant
Vmax: maximal velocity
Km: michaelis–menten constant
[I]: inhibitor concentration
