# Supplementary material for: Effect of bacteria type and sucrose concentration on levan yield and its molecular weight
Source: Microb Cell Fact. 2017 May 23;16:91. doi: 10.1186/s12934-017-0703-z (PMC5442672; doi:10.1186/s12934-017-0703-z)
Supplement: Supplementary file 1 — Additional file 1. Additional material A. [file 12934_2017_703_MOESM1_ESM.docx]

**Additional Material A**

**Glucose inhibition data**

| **TIEMPO** | **5 H** | Abs 400 nm | Abs 400 nm | Media | SD | [levan] | SD | dP/dt |
| --- | --- | --- | --- | --- | --- | --- | --- | --- |
|  | *Control* | 0.3007 | 0.3106 | 0.30565 | 0.0070 | 1.645 | -0.170 | 0.329 |
|  | *20* | 0.2678 | 0.2697 | 0.26875 | 0.0013 | 1.421 | -0.205 | 0.284 |
|  | *40* | 0.2394 | 0.2461 | 0.24275 | 0.0047 | 1.263 | -0.184 | 0.253 |
|  | *60* | 0.2125 | 0.2176 | 0.21505 | 0.0036 | 1.095 | -0.191 | 0.219 |
|  |  |  |  |  |  |  |  |  |
|  |  |  |  |  |  |  |  |  |
| **TIEMPO** | **8 H** | Abs 400 nm | Abs 400 nm | Media | SD | [levan] | SD | dP/dt |
|  | *Control* | 0.3901 | 0.3352 | 0.36265 | 0.0388 | 1.992 | 0.023 | 0.249 |
|  | *20* | 0.2919 | 0.296 | 0.29395 | 0.0029 | 1.574 | -0.195 | 0.197 |
|  | *40* | 0.2688 | 0.2772 | 0.273 | 0.0059 | 1.447 | -0.177 | 0.181 |
|  | *60* | 0.2442 | 0.2407 | 0.24245 | 0.0025 | 1.261 | -0.198 | 0.158 |

| **24 H** | Abs 400 nm | Abs 400 nm | Media | SD | [levan] | SD | dP/dt |
| --- | --- | --- | --- | --- | --- | --- | --- |
| *Control* | 0.4764 | 0.4578 | 0.4671 | 0.0132 | 2.627 | -0.133 | 0.109 |
| *20* | 0.385 | 0.3832 | 0.3841 | 0.0013 | 2.122 | -0.205 | 0.088 |
| *40* | 0.3342 | 0.3221 | 0.32815 | 0.0086 | 1.782 | -0.161 | 0.074 |
| *60* | 0.3019 | 0.3162 | 0.30905 | 0.0101 | 1.666 | -0.151 | 0.069 |

With the highest dP/dt values, dt/dS was estimated by using Vigants equivalence.

| **dP/dt** | **[I]** | **dt/dS** |
| --- | --- | --- |
| 0.3291 | 0 | 5.135877 |
| 0.2842 | 20 | 5.946631 |
| 0.2526 | 40 | 6.690854 |
| 0.2189 | 60 | 7.720217 |

If it is represented dt/dS vs. Glucose [I] concentration; the graph will be as follows:
