# Supplementary material for: Effect of bacteria type and sucrose concentration on levan yield and its molecular weight
Source: Microb Cell Fact. 2017 May 23;16:91. doi: 10.1186/s12934-017-0703-z (PMC5442672; doi:10.1186/s12934-017-0703-z)
Supplement: Supplementary file 2 — Additional file 2. Additional material B. [file 12934_2017_703_MOESM2_ESM.docx]

**Additional Material B**

**Economic study**

**Using juices factories byproducts as levan source**

**“Traditional” culture: without considering sucrose**

Yeast extract: 7 g·L^-1^. Price: 0.061€/g 🡪 0.427 €·L^-1^.

K_2_HPO_4_: 2.5 g·L^-1^. Price: 0.0004 €/g 🡪 0.001 €·L^-1^.

NH_4_SO_4_: 1.6 g·L^-1^. Price: 0.0988 €/g 🡪 0.158 €·L^-1^.

MgCl_2_: 0.4 g·L^-1^. Price: 0.0457 €/g 🡪 0.018 €·L^-1^.

Σ: 0.60 €·L^-1^

**“New” culture: without considering sucrose**

Byproduct from juice factory: 0 € 🡪 0 €·L^-1^

NH_4_NO_3_: 1 g·L^-1^. Price: 0.0868 €/g 🡪 0.0868 €·L^-1^

Difference between both medium: 0.08 €·L^-1^ and 0.60 €·L^-1^ (86%)

**If sucrose cost is included: 0.0293 €/g, and concentration of 150 gL^-1^**

Difference between both medium: 4.482 €·L^-1^ and 4.999 €·L^-1^ (10%). The save is 517 €·m^-3^
